# Blurring the lines: an empirical examination of the interrelationships among acceptability, appropriateness, and feasibility

**DOI:** 10.1186/s43058-024-00675-9

**Published:** 2024-12-18

**Authors:** Zoe Fehlberg, Zornitza Stark, Marlena Klaic, Stephanie Best

**Affiliations:** 1 Australian Genomics, Murdoch Children’s Research Institute, Melbourne, Australia; 2 School of Health Sciences, The University of Melbourne, Grattan Street, Melbourne, VIC 3010 Australia; 3 Victorian Clinical Genetics Services, Murdoch Children’s Research Institute, Melbourne, Australia; 4 The Royal Melbourne Hospital, Allied Health Department, Melbourne, Australia

**Keywords:** Implementation outcomes, Measurement instruments, Interrelationships, Mixed methods, Complex systems thinking, Implementation outcome proxy

## Abstract

**Background:**

Acceptability, appropriateness, and feasibility are established implementation outcomes used to understand stakeholders’ perceptions of an intervention. Further, they are thought to provide insight into behaviors, such as adoption. To date, measurement instruments for the three outcomes have focused on their individual assessment whilst nodding to the idea that they may interrelate. Despite this acknowledgment, there is little empirical evidence of the association among these constructs. Using the example of genetic health professionals providing additional genomic results to patients, this study aimed to examine the interrelationships among acceptability, appropriateness, and feasibility.

**Methods:**

A sequential explanatory mixed methods approach was employed. All genetic counsellors and clinical geneticists involved in a large research program were invited to complete pre/post surveys using existing measures of acceptability, appropriateness, and feasibility. Follow-up interviews, informed by the survey results, explored clinicians’ perspectives of the three outcomes in relation to providing additional genomic results to patients. To categorize interrelationships and generate feedback loops, survey data were analyzed using descriptive and correlation statistics and interpreted alongside interview data analyzed using content analysis.

**Results:**

The survey results (pre *n* = 53 and post *n* = 40) for each outcome showed a similar midpoint mean, wide ranges, and little change post implementation (Acceptability: pre *M* = 3.55, range 2–5 post *M* = 3.56, range 1.5–5; Appropriateness: pre *M* = 3.35, range 1–5, post *M* = 3.48, range 1–5; Feasibility: pre *M* = 3.30, post* M* = 3.32; range 1.25–5). The strength of correlation among outcomes ranged from 0.54 to 0.78. Five interrelationships were categorized from analysis of interview data (*n* = 14) and explain how clinicians’ perceptions of the intervention, positive or negative, were determined by interrelating factors of acceptability, appropriateness, and feasibility and that in different scenarios, the function and emphasis of importance among outcomes switched.

**Conclusions:**

Rather than existing separately, our study promotes the need to consider interrelationships among acceptability, appropriateness, and feasibility to better characterize clinicians’ perceptions of complex health care interventions and aid in the development of implementation strategies that have real world impact. Further, in the interest of reducing research waste, more research is needed to determine if the outcomes could serve as proxies for each other.

**Supplementary Information:**

The online version contains supplementary material available at 10.1186/s43058-024-00675-9.

Contributions to the literature • This study contributes empirical evidence about the nature of the interrelationships among the implementation outcomes of acceptability, appropriateness, and feasibility.

• Examining interrelationships takes a holistic approach to interpreting stakeholders’ perceptions and supports the growing attention on applying complex systems thinking to implementation science.

• The nature of the interrelationships may suggest that empirically, acceptability, appropriateness, and feasibility are indistinguishable and could serve as proxies for each other. In the interest of reducing research waste, future work is required to prioritize which outcome(s) could be enhanced and what are effective implementation strategies.

## Background

Acceptability, appropriateness, and feasibility are established implementation outcomes used to understand how stakeholders think and feel about an intervention or implementation strategy. They sit alongside a range of outcomes including, adoption, fidelity, implementation cost, penetration, and sustainability and are proposed as essential to assess implementation efforts [1]. Accepted definitions of the outcomes, albeit inconsistently used, [2] are that acceptability refers to “*stakeholders’ perceptions that an implementation target is agreeable, palatable, or satisfactory*” [1]. Appropriateness is the “*perceived fit, relevance, or compatibility of an implementation target for a given context, provider, consumer, or its perceived fit for a problem*” and Feasibility is the “*extent to which an implementation target can be successfully used or deployed within a given setting*” [1]. Categorized as ‘latent’ constructs, that is, although the outcomes cannot be directly observed, they provide valuable insight into predicting or explaining observable behaviors, such as adoption or fidelity [3, 4]. Vice versa, it is plausible that changes in adoption or fidelity are difficult to attain without targeting an aspect of acceptability, appropriateness, or feasibility [5]. However, there remains a dearth of evidence that links the outcomes to successful implementation strategies or to actual adoption or implementation [6]. Timing wise, the outcomes are thought to be more prominent during early phase implementation, with the updated Consolidated Framework for Implementation Research (CFIR) promoting antecedent assessment of the outcomes [7]. Importantly though, perceptions can shift and because of this dynamic process, iterative reassessment of the outcomes throughout implementation, over time, and in different context might be necessary [3]. Since many of the practice gaps implementation science aims to address relate to supporting changes in stakeholder behavior [8], generating robust methods to obtain stakeholders’ perceptions is critical to the field of implementation science.

### How are acceptability, appropriateness, and feasibility assessed?

Given the relationship between attitude and behavior, ‘adherence’, ‘uptake’, or ‘enrollment’ rates are frequently used to determine if stakeholders found an intervention ‘acceptable’ or ‘feasible’ [3, 9]. However, arguably behavior outcome data does little to understand the causes and has limited utility for designing solutions to address the problem. Alternatively, the outcomes are assessed by actively seeking stakeholders’ perceptions, utilizing an instrument to assist in the collection, assessment, and reporting [2]. One such instrument, developed by Weiner et al. [10], provides psychometrically validated, quantitative measures for acceptability, appropriateness, and feasibility (summarized in Table 1). With over 600 citations indexed in PubMed (on 9th April 2024), the Acceptability of Intervention Measure (AIM), Intervention Appropriateness Measure (IAM), and Feasibility of Intervention Measure (FIM) have been widely used predominately to capture clinician perceptions across a range of healthcare settings [11–14]. Owing to the pragmatic nature of the AIM, IAM, and FIM, the instruments frequently appear as part of implementation evaluations to determine if an innovation should be adopted, or if the implementation strategy trialed was effective. With no conventional threshold to proceed or demonstrated effectiveness established, higher scores are considered to indicate greater perceptions of acceptability, appropriateness, and feasibility.

**Table 1:** Summary of Acceptability (AIM), Appropriateness (IAM), and Feasibility (FIM) and measurement tools

**Outcome**	**Definition in literature** [1]	**Measures** [10]	**Items**^**a**^
Acceptability	*Stakeholders’ perceptions that an implementation target is agreeable, palatable, or satisfactory*	Acceptability of Intervention Measure (AIM)	Approve Appealing Like Welcome
Appropriateness	*The perceived fit, relevance, or compatibility of an implementation target for a given context, provider, consumer, or its perceived fit for a problem*	Intervention Appropriateness Measure (IAM)	Fitting Suitable Applicable Good-match
Feasibility	*The extent to which an implementation target can be successfully used or deployed within a given setting*	Feasibility of Intervention Measure (FIM)	Easy Doable Possible Implementable

^a^Each item is asked as a statement regarding the intervention being implemented

### What is known about the interrelationships among acceptability, appropriateness, and feasibility?

Although conceptually distinct, the way implementation outcomes play out in the real world may be less distinct [4, 6]. For example, as Prusaczyk et al. describe, although a provider may consider an intervention to be acceptable and appropriate to their role i.e., mental buy-in, they may still not find it feasible to adopt or implement because of any number of structural or contextual barriers [15]. Likewise, an intervention may be considered feasible and appropriate for a group of clinicians, but if it does not align with their personal ethics, an implementation problem relating to adoption or infidelity may arise. Positive improvements can also be imagined, whereby with time and practice a provider may find the intervention more feasible to provide and part of their professional identity, and hence will consider it acceptable to deliver. However, there is little known about the interactions between acceptability, appropriateness and feasibility, with only around 5% (21/400) of studies included in a 10-year review of Proctor et al.’s outcomes reporting interrelationships among any of the implementation outcomes [6]. The relationship between acceptability and appropriateness was identified in one study, acceptability and feasibility (*n* = 3), and appropriateness and feasibility (*n* = 1) [6]. No indication was provided in the review as to whether the included studies looked at the relationship among three or more outcomes, what the nature of the interrelationships was, or whether the interrelationship might differ for complex and simpler interventions.

Given the importance of acceptability, appropriateness, and feasibility in understanding the likelihood that an intervention will translate into day-to-day practice, it is critical to have useful measures that capture and articulate these outcomes collectively. Or, in the interest of reducing research waste, we need to identify if it is necessary to measure all three outcomes individually or whether any are interchangeable. Improvements to how we conceptualize, and measure acceptability, appropriateness, and feasibility could help focus attention to developing implementation strategies that lead to real world impact. With this in mind, we designed a study to examine interrelationships among acceptability, appropriateness, and feasibility.

## Methods

To examine possible interrelationships, we employed an explanatory sequential mixed method approach, whereby quantitative data were collected first and used to focus our qualitative enquiry, which in turn was used to elaborate on the quantitative findings (QUANT + qual). The approach to integrate the datasets during the interpretation stage of the study was selected as it provides a fuller picture of phenomenon [16].

Quantitative data were collected using the AIM, IAM, and FIM [10] deployed pre- and post- delivery of the program to establish clinician perspectives on acceptability, appropriateness, and feasibility of delivering a complex intervention (described below). We did not add any targeted implementation strategies to support implementation. Interviews were undertaken following the post- survey data collection, and towards the end of the program delivery. The survey data were used to inform the interview schedule which was designed to explore in-depth influences on clinicians’ perspectives of acceptability, appropriateness, and feasibility in relation to providing the intervention as part of routine practice.

### Research setting

**Fig. 1:**
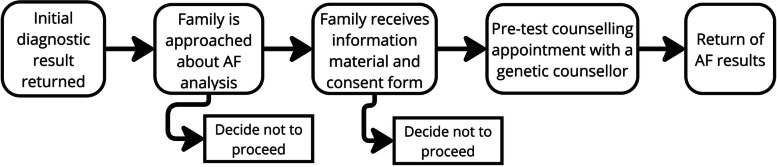
Pathway to families who consent to having additional findings analysis. Abbreviations: AF, Additional Findings. *Genetics Advisor is an online patient-facing digital health tool that can deliver genetics educational material such as pre-test counselling information [26]

The use of exome or genome sequencing (i.e., testing the entire genetic material for clinical purposes) in healthcare is expanding, and with it the possibility to report additional findings. Additional findings, are analyses of existing genomic data that are not related to the primary purpose of the genomic test but may have utility to an individual or family [17]. The provision of additional findings can be integrated into the initial diagnostic test, and differs between the patient selecting to opt-out of having additional findings such as in the USA [18], and models of care where patients choose to opt-in, such as in Canada [19] and European countries [20, 21]. In Australia, providing additional findings is not currently part of routine clinical practice [22]. This study was conducted within the Australian Acute Care Program [23, 24], which investigated the provision of genomic sequencing for infants and children who were admitted to intensive care with a suspected genetic condition. Within the program, additional findings were offered to families, described here [25]. Briefly, around three to six months after the initial diagnostic result, families were offered the choice of additional analyses of their genomic data for: 1) pediatric-onset treatable and non-treatable conditions in the child (e.g., muscular dystrophy); 2) adult-onset ‘actionable’ conditions in the parents (e.g., genetic heart conditions); and 3) reproductive carrier screening in the parents (e.g., carrier for cystic fibrosis). Figure 1 depicts the pathway for families who consented to receive additional findings.

### Participants and recruitment

All clinical geneticists (including trainees) and genetic counsellors involved in the Acute Care Program were eligible to participate (54 clinical geneticists/fellows and 44 genetic counsellors were employed from 17 tertiary or children’s hospitals across Australia) [27]. Survey invitations were sent via email from the study lead [Z.S.] with one reminder. The same approach was used to send the first interview invitation, with follow-up invitations sent to a select group using purposive sampling to ensure national representation from each of the six study site jurisdictions [28].

The study was approved by the Royal Children’s Hospital Melbourne, Human Research Ethics Committee (HREC/71973/RCHM-2023). After the opportunity to view study information, survey participants could click to consent and proceed, and interview participants provided verbal consent prior to the interview commencing.

### Data collection tools and procedure

#### Survey

The surveys were anonymous, available for two weeks (pre-survey deployed in May 2021 and the post-survey in April 2023) and hosted online using REDCap (Research Electronic Data Capture) platform [29]. The surveys were expected to take < 5 min and included brief demographic questions followed by the AIM, IAM and FIM measures (Table 1) which have four items per outcome and require a response on a 5-point Likert scale ranging from completely disagree to completely agree. An example of the survey instrument is provided in Additional File 1.

#### Interview

Semi-structured interviews were conducted towards the end of the program (June 2023) and were guided by an interview schedule that was deliberatively designed with questions to explicitly address each of the three outcomes individually, as they all scored similarly in the survey (Table 2). Following pilot testing, changes were made to improve the discriminant validity of the interview schedule. Additional clarity around the definitions of the outcomes were added at the beginning of the interview and as a preface to each question. The questions were also re-ordered to reflect how the outcome scored in the survey, as it was thought the poorest scoring outcome might be at the forefront of participants’ minds. The complete interview schedule is provided in Additional File 2. To maintain consistency, interviews were conducted by one researcher [Z.F.] via Zoom videoconference at the participants’ convenience and were expected to take around 30–45 min. Audio-recordings were transcribed and de-identified by the study team with the assistance of the Zoom transcribe feature.

**Table 2:** Interview questions specific to the outcome of interest

Outcome	Interview text—Outcome preface and question
Feasibility	*The first outcome of interest is ****feasibility**** which is to do with the extent to which something can be successfully used or deployed within a given setting* **Interview Question:** *How feasible would you (your department) find incorporating additional findings into routine practice?*
Appropriateness	*The next outcome is ****appropriateness**** which is to do with the perceived fit, relevance, or compatibility of an implementation target for a given context, provider, consumer, or its perceived fit for a problem* **Interview Question:** *How would incorporating additional findings into routine practice fit within your current day-to-day practice?*
Acceptability	*The final outcome is ****acceptability**** which is around perceptions that an implementation target is agreeable, palatable, or satisfactory* **Interview Question:** *With this in mind, how would you feel about providing additional findings to families as part of routine practice?*

### Data analysis

#### Survey

Survey data were analyzed using descriptive statistics to summarize demographic characteristics. The four items per measure were summed and a mean score calculated. Individual participant responses were also plotted on a line graph. To assess the interrelationships, Spearman’s rank-order correlation were computed among outcomes with a higher score demonstrating correlation (1 being perfect, 0.9–0.7 being strong, 0.6–0.4 being moderate, 0.3–0.1 being weak and 0 being none) [30]. Bonferroni correction was used with *P* values for statistical significance set at < 0.05. Data were analyzed in STATA SE18.

#### Interview

First, interview data were analyzed using a deductive content analysis approach, [31] whereby segments of text relating to the implementation outcome were color-coded using Acceptability = yellow, Appropriateness = blue, and Feasibility = green. This step was guided by a codebook, containing the definition of the outcome given by Proctor et al., [1] the Weiner scale items, [10] and a generated definition in context, provided in Additional File 3. One researcher color-coded all the transcripts [Z.F.] with challenging segments discussed at regular meetings with [S.B.] who double coded three transcripts.

Following the color-coding, which facilitated in-depth familiarization with the data, an inductive content analysis approach was used to categorize interrelationships [32]. One researcher [Z.F.] created preliminary interrelationships by grouping printed-out and cut-up sections of the color-coded transcripts where similar patterns of meaning could be interpreted. The preliminary groupings of interrelationships were then reviewed, reconceptualized, and refined in a session with [S.B.], before a team discussion with [Z.S. and M.K.] to ensure that the interrelationships developed comprehensively and concisely captured what was meaningful about the data and reflected the central ideas. The team continued to refine the framing of the interrelationships, including during the writing up of the results. Feedback loops were designed to visually represent scenarios that indicate the nature of the interrelationships.

#### Integration of quantitative and qualitative data

Data were mixed at two timepoints. First, the quantitative data informed the design of the interview schedule. Then during interpretation of the datasets, analysis of the qualitative data provided detailed exploration of the findings of the survey data by looking for ways to categorize explanations for the correlations of the three implementation outcomes [16].

## Results

### Characteristics of participants

#### Survey

Fifty-three clinicians (clinical geneticists or trainees *n* = 33, 62% and genetic counsellors *n* = 20, 37%) completed the pre survey (response rate: 53/98, 54%). Forty clinicians (clinical geneticists or trainees *n* = 20, 50% and genetic counsellor *n* = 20, 50%) completed the post survey (response rate: 40/77 52%), accounting for the 21 clinicians who were no longer directly involved in the study.

#### Interview

Of the clinicians invited to an interview, 15 responded and 14 were interviewed, as one individual was no longer available. Participants were mostly genetic counsellors (*n* = 12, 86%) as they were the predominant clinicians involved in providing additional findings. The participants represented each of the six study sites, reflective of the size of the patient population (Site 1 *n* = 5, Site 2 *n* = 2, Site 3 *n* = 4, Site 4 *n* = 1, Site 5 *n* = 1, Site 6 *n* = 1). The interviews ran for an average of 35 min (range 23–50).

### Interrelationships among acceptability, appropriateness, and feasibility

#### Survey

**Fig. 2:**
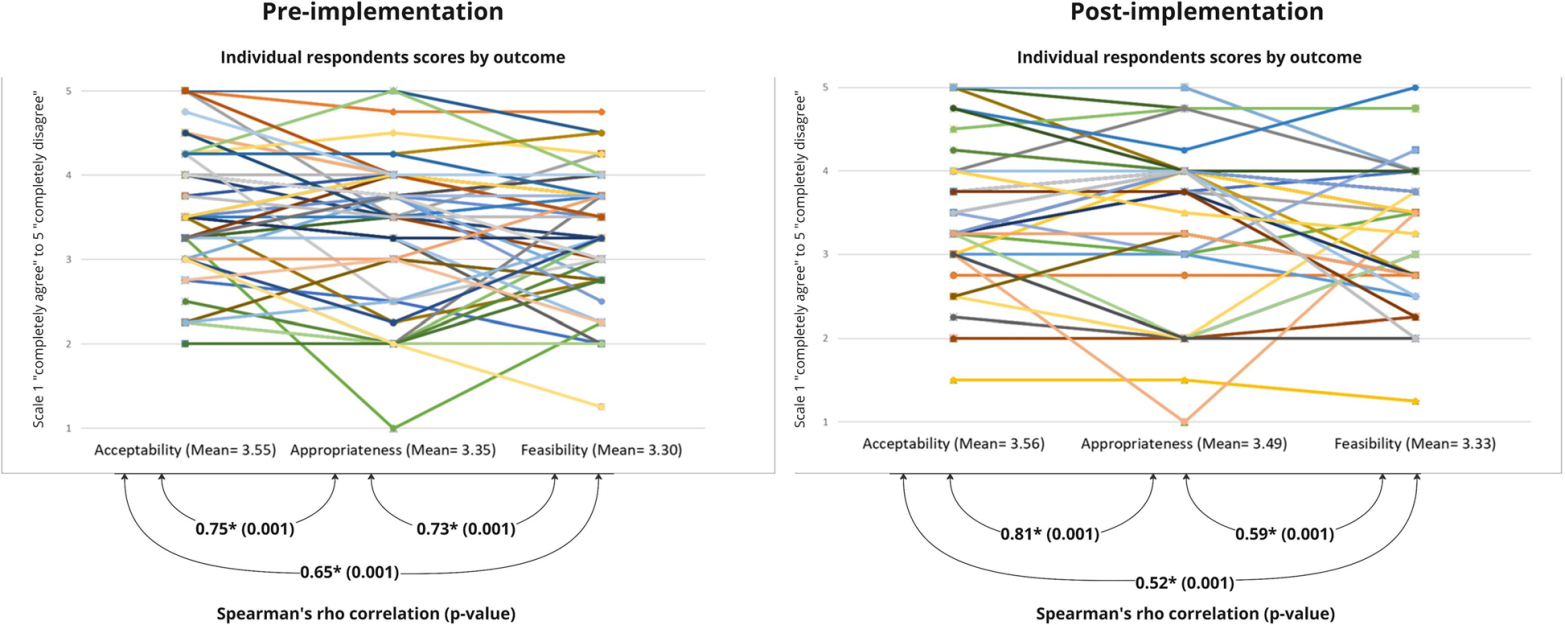
Pre (*n* = 53) and Post (*n* = 40) mean implementation outcome scores by individual respondent and correlation as Spearman’s rho (*P* Value)

Overall, the acceptability, appropriateness, and feasibility of providing additional findings pre- implementation were classified with borderline mean scores “neither agree nor disagree” (Acceptability (AIM) *M* = 3.55; range 2–5), (Appropriateness (IAM) *M* = 3.35; range 1–5), (Feasibility (FIM) *M* = 3.30; range 1.25–4.75), with little change post implementation (Acceptability (AIM)* M* = 3.56; range 1.5–5), (Appropriateness (IAM) *M* = 3.48; range 1–5), (Feasibility (FIM) *M* = 3.32; range 1.25–5). However, Fig. 2 plots individual responses and shows the variation of individual participant responses between outcomes and that there were significant correlations among all outcomes with strength ranging from strong 0.81 to moderate 0.52. Post implementation, acceptability and appropriateness remained strongly correlated, whereas the strength of the correlation between acceptability and feasibility and appropriateness and feasibility decreased in moderation (Pre 0.65 vs Post 0.52 and Pre 0.73 vs Post 0.59, respectively).

#### Interview

**Fig. 3:**
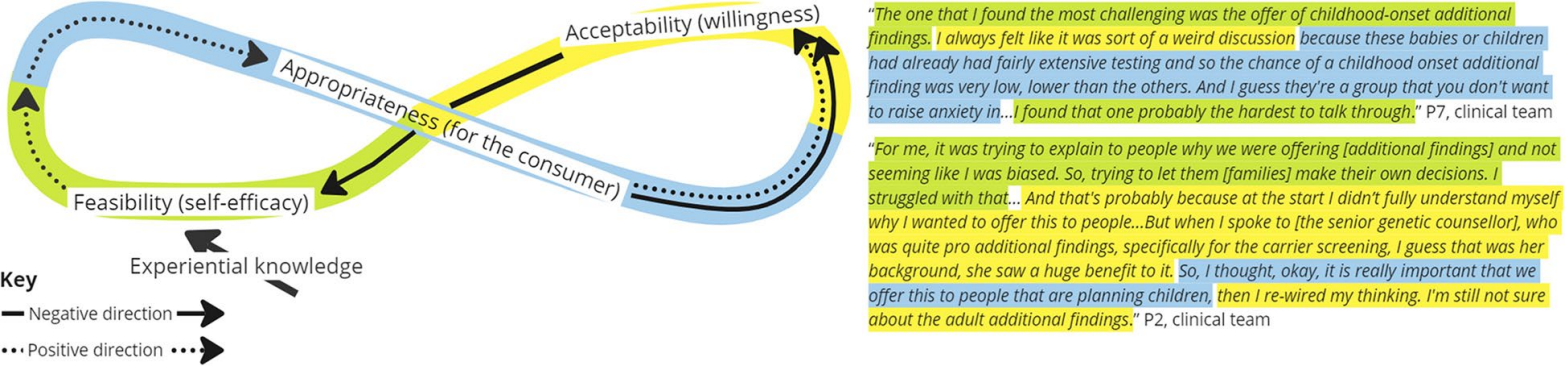
Interrelationship #1: Clinician perception of appropriateness influences acceptability and self-efficacy

**Fig. 4:**
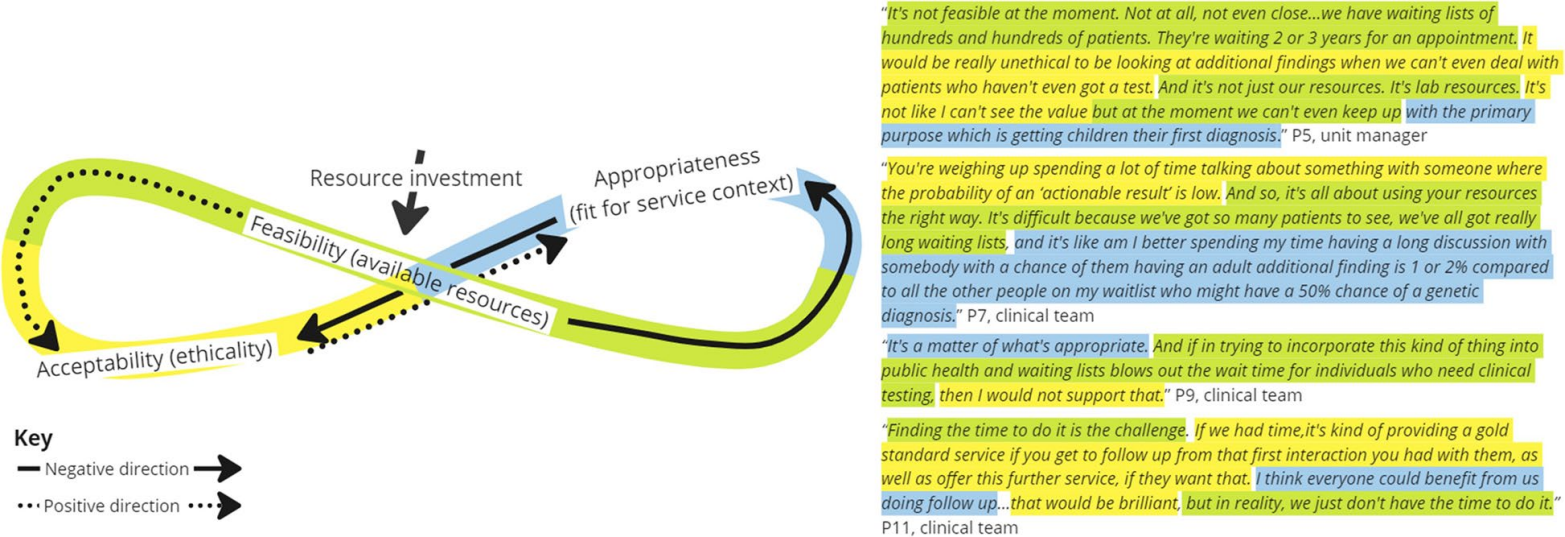
Interrelationship #2: Weighing up the possible utility versus the resources required and impact on existing clinical practice

**Fig. 5:**
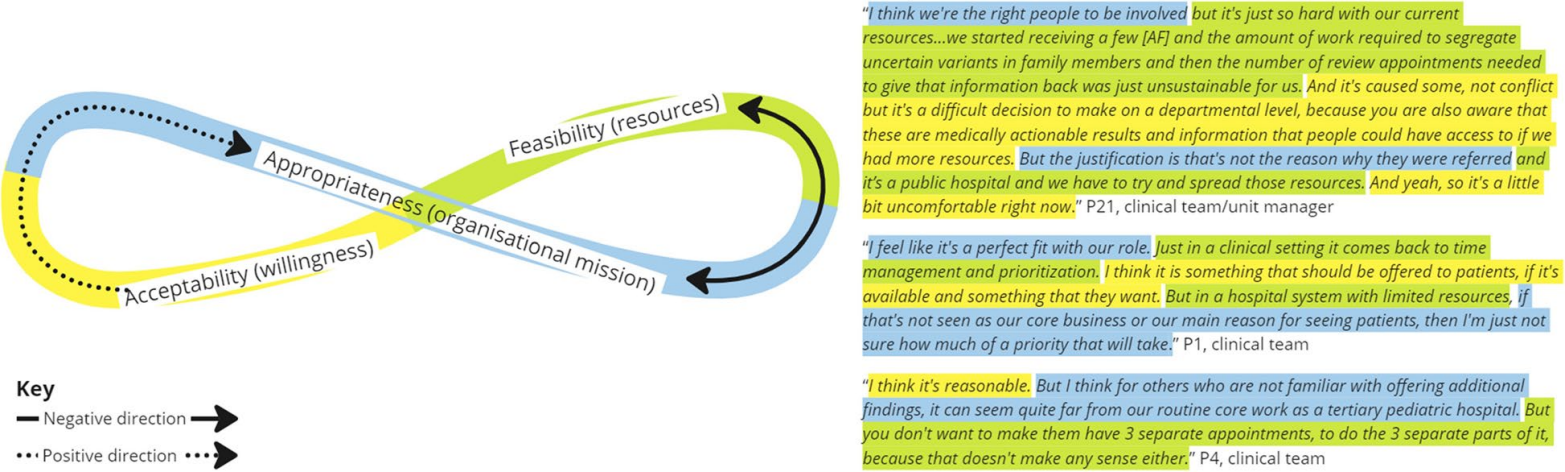
Interrelationship #3: Organizational boundaries in relation to resourcing regardless of acceptability

**Fig. 6:**
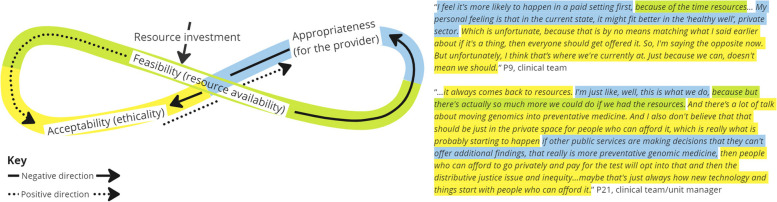
Interrelationship #4: Equity in service provision in relation to availability of resources for a given setting

**Fig. 7:**
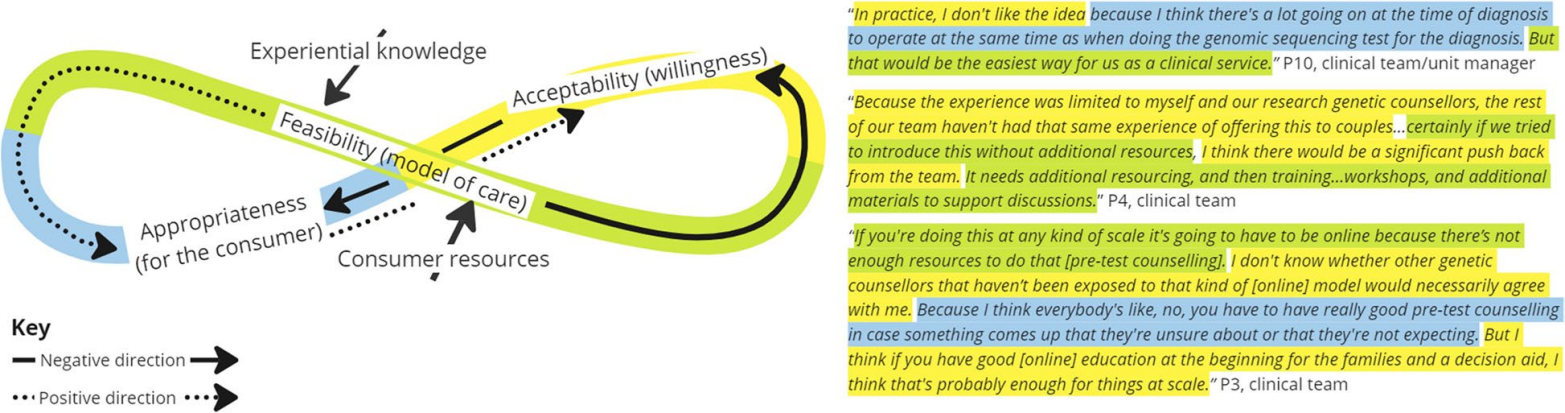
Interrelationship #5: Acceptability towards integrating the innovation into routine practice in relation to clinician capacity, current workflows, and consumer centeredness

Five interrelationships were categorized from analysis of the interview data and are described below using exemplary scenarios and feedback loops displayed in Figs. 3, 4, 5, 6 and 7. Each figure of 8 loop is designed to visualize the intertwining and complex nature of the interrelationships and how in different scenarios the central influencing implementation outcome changes. The Figures can be interpreted by beginning in the center and following the direction of the arrows, whilst noting how interjections e.g., ‘resource investment’ can positively alter the direction, example quotes are presented alongside each figure.

##### Interrelationship #1 feasibility, acceptability, and appropriateness. A clinician’s perception of self-efficacy influences acceptability and perceived appropriateness for the consumer (Fig. 3)

A three-way interrelationship where clinicians’ perceptions of how easy (feasible) and acceptable (willingness) the intervention is to deliver is lowered because of the perceived appropriateness for the consumer group. Also evident is how experiential knowledge improves feasibility, which has a positive influence on acceptability and appropriateness i.e., the more you do it the easier it gets and the more you recognize the benefits. The interrelationship demonstrates how implementation strategies that encourage learning by doing can have positive impacts on perceptions of acceptability and appropriateness.

##### Interrelationship #2 acceptability, feasibility, and appropriateness. Weighing up the possible utility versus the resources required and impact on existing clinical practice (Fig. 4)

Here, a three-way interrelationship shows how perceptions of acceptability (ethicality) are lowered because of feasibility (time and resources constraints) and the consequences that implementing the intervention will have on providing an existing service in comparison to the benefit (appropriateness). The interrelationship demonstrates how ‘implementation cost’ in terms of both financial and opportunity costs, i.e., I will have to give up other activities to deliver the intervention, influences perceptual outcomes of feasibility, acceptability, and appropriateness.

##### Interrelationship #3 appropriateness, feasibility, and acceptability: organizational boundaries in relation to resourcing regardless of acceptability (Fig. 5)

The interrelationship shows that although an intervention might be acceptable to, and appropriate for the clinical role, if at a service level the intervention is not seen as ‘core business’ or a priority (fit for organization mission) then implementation is less feasible due to resource constraints (feasibility). No positive influences were described by participants and demonstrates the need for strategies that not only target individuals but also the organization and service system.

##### Interrelationship #4 acceptability, feasibility, and appropriateness: equity in service provision in relation to availability of resources for a given setting (Fig. 6)

This interrelationship shows how perceptions of who is an appropriate service provider are influenced by the availability of resources and that these considerations also raise ethicality concerns which influence perceptions of acceptability.

##### Interrelationship #5 acceptability, feasibility, and appropriateness: acceptability towards integrating the innovation into routine practice in relation to clinician capacity, current workflows, and consumer centeredness (Fig. 7)

The interrelationship shows a tension between a feasible and appropriate model of care and how that influences perceived acceptability. Prior experience and perceptions of clinical role, was seen to improve clinician acceptability towards a streamlined model of care with added inbuilt resources for clinicians and consumers.

## Discussion

With relatively poor sustainment of healthcare interventions beyond the initial implementation phase, [33] increased attention to constructs that predict or explain behavior, such as acceptability, appropriateness, and feasibility, is required. Although formative research has established conceptually distinct implementation outcomes and measurement tools, our findings demonstrate that acceptability, appropriateness, and feasibility, do not play out in isolation and separating them may contribute to health research waste and/or risk foregoing valuable implementation insights.

One interpretation of the findings is that a holistic approach to examining implementation outcomes and their interrelationships may need to be considered and supports the burgeoning attention of applying complex systems thinking to implementation science e.g., [34–37]. Complex systems thinking focuses on the features and function of the system as a whole (inter-relations, dynamism, emergencies, unpredictability, feedback loops, dependencies, and all) and calls for research designs and methods to better reflect the complex system in which an intervention is placed [34]. Applying a complex systems lens, acknowledges that a sequential implementation pipeline fails to capture the intricacies of complex systems [38, 39], and our findings indicate the idea may also apply to the assessment of implementation outcomes [9]. The five interrelationships exemplified through the study depict dynamic feedback loops which visualize how the order and emphasis of importance of outcomes can switch depending on influential tendencies such as self-efficacy (interrelationship #1) or available resources (interrelationships #2–4).

Going forward, incorporating aspects of complex system thinking, such as understanding that although individually these outcomes are important and using deliberative measures to understand them is useful, their meaning and implications are bigger than the sum of their parts. To harmonize complex systems thinking with implementation science necessitates both research designs and methods to work within the complexity of the health care system, and have measurement tools that can capture the interactions between implementation outcomes [40]. Value could be added to measurement tools, such as the AIM, IAM, and FIM, if they could be used to understand mechanistic trade-offs, thresholds, and tolerances among implementation outcomes and generate evidence as to how much effort is needed and when to see improvements.

Another interpretation, is that during the psychometric assessment of the AIM, IAM, and FIM, Weiner et al., suggest that if two or more of the outcomes are correlated, then perhaps they could serve as proxies for each other [10]. The nature of the interrelationships categorized from our data indicate that acceptability, appropriateness, and feasibility are associated. The survey results showed very little difference between the mean scores of each outcome and strong to moderate correlation scores. Despite deliberate efforts during interviews to focus on a specific implementation outcome by asking directed questions, and using accepted definitions, participant responses frequently intertwined two or all outcomes. Indeed, our stakeholders, in this case, clinicians, were cognizant of interrelationships among outcomes and the challenges to disentangling them, as one participant put it, “*there’s a Venn diagram and it [implementation outcomes] definitely overlaps*” (P9, clinical team). However, our survey results showed that the correlation between appropriateness and feasibility trended downwards, suggesting that overtime our participants were able to better differentiate between the appropriateness for the setting and the perceived ease of implementation.

Deciding which outcome may serve as a fitting proxy for another would form part of a future research agenda, however, our findings suggest that acceptability could likely be a favorable proxy for appropriateness and feasibility. For example, interrelationships #2 demonstrates how clinicians use feasibility and appropriateness to justify their perceived acceptability. Acceptability is the most frequently assessed outcome of the three [2, 6] and has comprehensive frameworks and data collections tools, such as the Theoretical Framework of Acceptability (TFA) [5]. The TFA consists of seven constructs: affective attitude, burden, perceived effectiveness, ethicality, intervention coherence, opportunity costs, and self-efficacy, of which most featured in our data. These seven constructs provide a more comprehensive picture of acceptability, compared to the AIM ‘acceptability’ items (like, welcome, approve, and appeal) which would map to understanding the TFA construct of affective attitude. Further, other TFA constructs such as ‘burden’ conceptually overlap with the FIM ‘feasibility’ items (easy, doable, possible, implementable).

Future enhancement of how acceptability is theorized and applied will be required, including establishing implementation strategies that effectively target constructs within the TFA. Encouragingly, if the outcomes are related, then efforts to improve acceptability would most likely see changes in how clinicians perceived the others. For example, although resource constraints were raised by our participants as a significant feasibility barrier, enablers beyond increased resourcing were raised, such as exposure to and experiential knowledge of a less time intensive ‘hands-off’ model of care that make use of digital platform. Further work should also be undertaken to understand if the TFA and other implementation outcome measures translate across diverse populations.

Finally, we may need to rethink how early implementation outcomes are bundled, for example, with readiness and equity. There is a growing evidence-base to suggest that *implementation readiness* is a critical part of implementation [41] and a precursor to early implementation success [42]. This assumption is reflected in the clustering of outcomes within the updated Consolidated Framework for Implementation Research (CFIR) [7]. Readiness includes concepts of *willingness* and *ability* [43] which conceptually involves aspects of acceptability and feasibility that were fore fronted in our findings. Therefore, it may be plausible that acceptability, appropriateness, and feasibility are subconstructs of perceived individual, organizational, and system readiness. Our findings suggest implementation outcomes can also signal equity considerations [6]. Many of our interview participants emphasized how their perceptions of the intervention appropriateness and acceptability were lowered because of possible inequitable access in service provision. We propose the boundaries between implementation outcomes and other factors such as equity are fuzzy, and that producing feedback loops are flexible enough to seamlessly incorporate additional considerations, including service outcomes.

### Future research directions

All three interpretations of our study require empirical investigation that may include the following research questions.

1. How can applying complex systems thinking to the measurement of implementation outcomes support the development of implementation strategies?
2. Which of the outcomes could serve as a suitable proxy for other outcomes by providing the most comprehensive assessment of stakeholder’s perceptions that predict behavior, and does this depend on additional factors such as the complexity of the intervention?
3. What determinants of implementation should be considered for bundling with which implementation outcomes and in what contexts.

### Limitations

A limitation of this study was that our findings related to one complex intervention and therefore necessitate application to other settings and populations. For example, we sought the perspectives of clinicians; however, we acknowledge the importance of including other stakeholders such as policy makers, consumers, and researchers. Even though the survey required minimal effort (< 5 min to complete), the response rate was around 50% and therefore may not reflect the viewpoints of the entire study population. Further the survey sample was not paired, which limits pre and post inferences and the ability to estimate correlations among outcomes overtime and is an important future research direction. The interrelationships focused on early phase implementation and are unlikely exhaustive, or necessarily translate to other stages of implementation, however, they begin to explain why correlation between outcomes were observed in the survey findings. Our participants worked within the Australian public health system and our findings may not resonate with other health system structures and cultural contexts.

## Conclusions

Determining how to optimize the examination of outcomes that influence stakeholder behavior is essential to supporting sustained implementation of interventions beyond the research setting. Rather than existing separately, our study promotes the need to consider interrelationships among acceptability, appropriateness, and feasibility to better characterize clinicians’ perceptions of complex health care interventions and aid in the development of implementation strategies that have real world impact. Further, in the interest of reducing research waste, more research is needed to determine if the outcomes could serve as proxies for each other.

## Supplementary Information

Additional file 1: Example Survey. This file contains an example of the survey tool using the AIM, IAM, FIM.

Additional file 2: Interview Guide. This file contains the interview schedule used in this study.

Additional file 3: Coding guide. This file contains the coding guide for each implementation outcome with definition in literature (Proctor et al. 2011 1 ) the AIM, IAM, and FIM measure items (Weiner et al. 2017 2 ), and context specific definitions.

## Data Availability

The datasets used and/or analyzed during the current study are available from the corresponding author upon request.

## Abbreviations

AIM: Acceptability of Intervention Measure
IAM: Intervention Appropriateness Measure
FIM: Feasibility of Intervention Measure
GC: Genetic Counsellor
CG: Clinical Geneticist
TFA: Theoretical Framework of Acceptability
CFIR: Consolidated Framework for Implementation Research
