# Supplementary material for: Blurring the lines: an empirical examination of the interrelationships among acceptability, appropriateness, and feasibility
Source: Implement Sci Commun. 2024 Dec 18;5:139. doi: 10.1186/s43058-024-00675-9 (PMC11657390; doi:10.1186/s43058-024-00675-9)
Supplement: Supplementary file 1 — Additional file 1: Example Survey. This file contains an example of the survey tool using the AIM, IAM, FIM. [file 43058_2024_675_MOESM1_ESM.pdf]

Additional Material 1: Example Survey

1. Acceptability i.e., how acceptable you feel it is to offer analysis for additional findings from existing genomic data.

	Completely disagree	Disagree	Neither agree nor disagree	Agree	Completely agree
1. I approve of offering additional findings.	○	○	○	○	○
2. Offering additional findings is appealing to me.	○	○	○	○	○
3. I like offering additional findings.	○	○	○	○	○
4. I welcome offering additional findings.	○	○	○	○	○

2. Appropriateness i.e., how offering analysis for additional findings fits or is compatible with your current day to day practice

	Completely disagree	Disagree	Neither agree nor disagree	Agree	Completely agree
1. Offering additional findings seems fitting.	○	○	○	○	○
2. Offering additional findings seems suitable.	○	○	○	○	○
3. Offering additional findings seems applicable.	○	○	○	○	○
4. Offering additional findings seems like a good match with my current day to day practice.	○	○	○	○	○

3. Feasibility i.e., whether analysis for additional findings can be offered successfully in your work setting

	Completely disagree	Disagree	Neither agree nor disagree	Agree	Completely agree
1. Offering additional findings seems implementable.	○	○	○	○	○
2. Offering additional findings seems possible.	○	○	○	○	○
3. Offering additional findings seems doable.	○	○	○	○	○
4. Additional findings seems easy to offer.	○	○	○	○	○

4. (optional) Please add any further comments you may have about your thoughts on the acceptability, appropriateness, and feasibility of offering additional findings:
