# Supplementary material for: Blurring the lines: an empirical examination of the interrelationships among acceptability, appropriateness, and feasibility
Source: Implement Sci Commun. 2024 Dec 18;5:139. doi: 10.1186/s43058-024-00675-9 (PMC11657390; doi:10.1186/s43058-024-00675-9)
Supplement: Supplementary file 2 — Additional file 2: Interview Guide. This file contains the interview schedule used in this study. [file 43058_2024_675_MOESM2_ESM.pdf]

Additional Material 2: Interview Guide

The Acute Care Program – Additional Findings (AF)

Interview schedule

Opening:

Thank you for your time today. We are running this series of interviews because, as you are probably aware, there is increased discussion about the provision (or not) of AF following genomic sequencing for patients and families. The patients' perspective is essential and is being covered through a separate set of interviews. Equally important is the perspectives of those delivering the service. The aim of these interviews is to learn from healthcare practitioners like yourself who have been involved in the AF program.

Interview structure: To give you a bit of a sense of how things will run, first I will ask your thoughts on the model of care or the process that was used to deliver the AF study and then I'll ask you about your thoughts about future delivery. Throughout, there are three outcomes that we are particularly interested in that you might be familiar with from the survey. We will talk about the **feasibility** or the extent to which AF can be successfully implemented, **appropriateness** which is more to do with the fit or relevance of providing additional for you work and within your workplace, and **acceptability** which is around perceptions of how you feel towards incorporating AF into routine care.

Interview Schedule Questions

**Area of interest**	**Sample questions**
**Context and experience**	To start off, could you please tell me a little bit about your role and whether you had any prior experience with providing additional findings?<br><br>What about through the study, what was your experience with providing additional findings?<br><br>Prompt- Did you need to undertake any additional training? How did you find this?
**Intervention**	How did you find providing additional findings through the study?<br><br>Were there any challenges you can speak of to providing additional findings?<br><br>On the flipside – was there anything helped you provide additional findings?<br><br>Where there any unexpected impacts or consequences on your work (or the department) from providing additional findings?
**Future –** Moving onto thinking about incorporating additional findings within routine practice	
**Feasibility**	The first outcome of interest is **feasibility** which is to do with the extent to which something can be implemented<br><br>*Question asked:* How would you (your department) find incorporating AF into routine practice?

**Appropriateness**	The next outcome is **appropriateness** which is to do with the relevance of implementing something for your work and or within your workplace<br><br>How would incorporating AF into routine practice fit with your current day-to-day practice?
**Acceptability**	The final outcome is **acceptability** which is around perceptions of how you feel about implementing something<br><br>How do you feel towards incorporating AF into routine practice?
	How, if at all do you see AF being delivered in the future?<br><br>What do you think will be the biggest barriers to providing AF as part of routine care?<br><br>Is there anything more that could be done to improve the process of delivering AF for you (your department)?<br><br>Is there anything you have learnt from providing AF that you would share with others?

Closing: Is there anything else we haven't covered that you would like to share?
