# Supplementary material for: Blurring the lines: an empirical examination of the interrelationships among acceptability, appropriateness, and feasibility
Source: Implement Sci Commun. 2024 Dec 18;5:139. doi: 10.1186/s43058-024-00675-9 (PMC11657390; doi:10.1186/s43058-024-00675-9)
Supplement: Supplementary file 3 — Additional file 3: Coding guide. This file contains the coding guide for each implementation outcome with definition in literature (Proctor et al. 2011 1 ) the AIM, IAM, and FIM measure items (Weiner et al. 2017 2 ), and context specific definitions. [file 43058_2024_675_MOESM3_ESM.pdf]

Additional Material 3: Coding guide for each implementation outcome with definition in literature (Proctor *et al* 2011[1]) the AIM, IAM, and FIM measure items (Weiner *et al* 2017[2]), and context specific definitions.

**Implementation outcome**	Definition in literature (Proctor[1] and Weiner[2] items)	Definition in context
**Acceptability** (yellow)	*Proctor: Stakeholders' perceptions that an implementation target is agreeable, palatable, or satisfactory*. AIM items: *Approve, Appealing, Like, Welcome*	Clinician perceptions that providing additional findings is agreeable, palatable, or satisfactory.
**Appropriateness** (blue)	Proctor*: The perceived fit, relevance, or compatibility of an implementation target for a given context, provider, consumer, or its fit for a problem* IAM items: *Fitting, Suitable, Applicable, Good match*	Clinician perceptions towards the perceived fit, relevance or compatibility of providing additional findings within a) a paediatric genetics service, b) as clinician, c) for families who have had whole genome sequencing; or d) perceived fit of additional findings to address a particular issue.
**Feasibility** (green)	Proctor*: The extent to which an implementation target can be successfully used or deployed within a given setting*. FIM items: *Implementable, Possible, Doable, Easy*	Clinician perceptions towards the extent to which providing additional findings can be successfully used or deployed within a given setting.
